# Characterization of the complete chloroplast genome of *Aconitum pendulum* (Ranunculaceae), an endemic medicinal herb

**DOI:** 10.1080/23802359.2019.1703592

**Published:** 2020-01-08

**Authors:** Ze-Huan Wang, Ya-Qiong Li

**Affiliations:** Faculty of Traditional Chinese Pharmacy, Yunnan University of Traditional Chinese Medicine, Kunming, China

**Keywords:** *Aconitum pendulum*, complete chloroplast genome, phylogenetic analysis, Ranunculaceae

## Abstract

*Aconitum pendulum* is an endemic medicinal herb, which has effects of relieving pains and is widely used to treat frostbite, rheumatoid arthritis, and so on. In this study, we sequenced the complete chloroplast genome with Illumina sequencing technology. The complete chloroplast genome length is 155,597 bp, shows a typical tetrad structure, which manifests as one large and one small single copy (LSC and SSC) regions of 86,336 and 16,961 bp, isolated by two inverted repeat regions (IRs) of 26,150 bp. This study annotated altogether 131 unique genes, consisting of 86 protein-encoding genes, 8 rRNA, and 37 tRNA. According to the maximum likelihood phylogenetic tree based on 20 complete chloroplast genomes, *A. pendulum* shows close association with additional *Aconitum* subgenus. The chloroplast genome-wide for *A. pendulum* would help to conserving the precious natural populations.

The Ranunculaceae *Aconitum* L. covers nearly 400 species in the northern hemisphere across the temperate regions. It is a perennial or pseudoannual genus with an erect or twining stem and blue, purple, or yellow flowers (Li and Kadota 2001). In China, approximately 76 *Aconitum* species are utilized to be the medicinal plants to treat diverse pain types and rheumatoid arthritis (RA) (Xiao et al. 2006). Typically, *Aconitum pendulum* Busch. has been recognized as the endemic medicinal perennial herb, which is distributed in southwest and northwest China. *A. pendulum* is an endemic medicinal herb, the tuberous mother root has been used to treat frostbite, RA, and so on (Commission of Chinese Pharmacopoeia 2015). The natural habitat and population of this species have been extremely reduced by human activities. Therefore, it is necessary for us to learn more about its genetic data and pay more attention to it. Notably, the chloroplast genome-wide for *A. pendulum* would help to conserve the precious natural populations.

In this study, silica-gel-dried leaves of *A. pendulum* were collected from Xianggelila of Yunnan Province, China (99°44.292′E, 27°41.583′N), and voucher specimens (AP201808001) were deposited in the Herbarium of Yunnan University of Chinese Medicine. Total genomic DNA was extracted with the CTAB method (Doyle and Doyle 1987). We sequenced the complete chloroplast genome with Illumina Hiseq X-Ten (Illumina, San Diego, CA, USA), reads of the complete chloroplast genome were assembled using de novo assembling constructed in SPAdes 3.9.1 (Bankevich et al. 2012), using kmer lengths of 21–105 bp, followed by reference guided assembling conducted with Bandage 0.8.1 (Wick et al. 2015) and Geneious 9.1.4 (Kearse et al. 2012). *Aconitum delavayi* (NC_038097) was used as reference for annotation using GeSeq (Tillich et al. 2017), coupled with manual correction for boundaries. The circular chloroplast genome map was drawn using the OGDRAW program (Greiner et al. 2019). To identify the phylogenetic position of *A. pendulum*, the maximum likelihood (ML) tree was reconstructed based on 20 species complete chloroplast genomes by MEGA X (Kumar et al. 2018).

The complete chloroplast genome of *A. pendulum* was 155,662 bp in length (GenBank accession number MN719135), the GC content was 38.1%. LSC and SSC contained 86,384 bp and 16,972 bp, respectively, while IR was 26,153 bp in length. A total of 131 unique genes were annotated, including 37 tRNA, 8 rRNA, and 86 protein-coding genes. Seven protein-coding genes, seven tRNA, and four rRNA genes were duplicated in the IR regions. In total, 17 intron-containing genes were in the chloroplast genome of *A. pendulum* of which two genes (clpP and ycf3) include two introns and the rest include a single intron.

Phylogenetic trees show that *A. pendulum* and other *Aconitum* subgenus. *Aconitum* species formed a monophyletic clade with 100% bootstrap support value (Figure 1). The complete chloroplast genome of *A. pendulum* would help to understanding the genetic information and conserving the precious natural populations.

**Figure 1.**
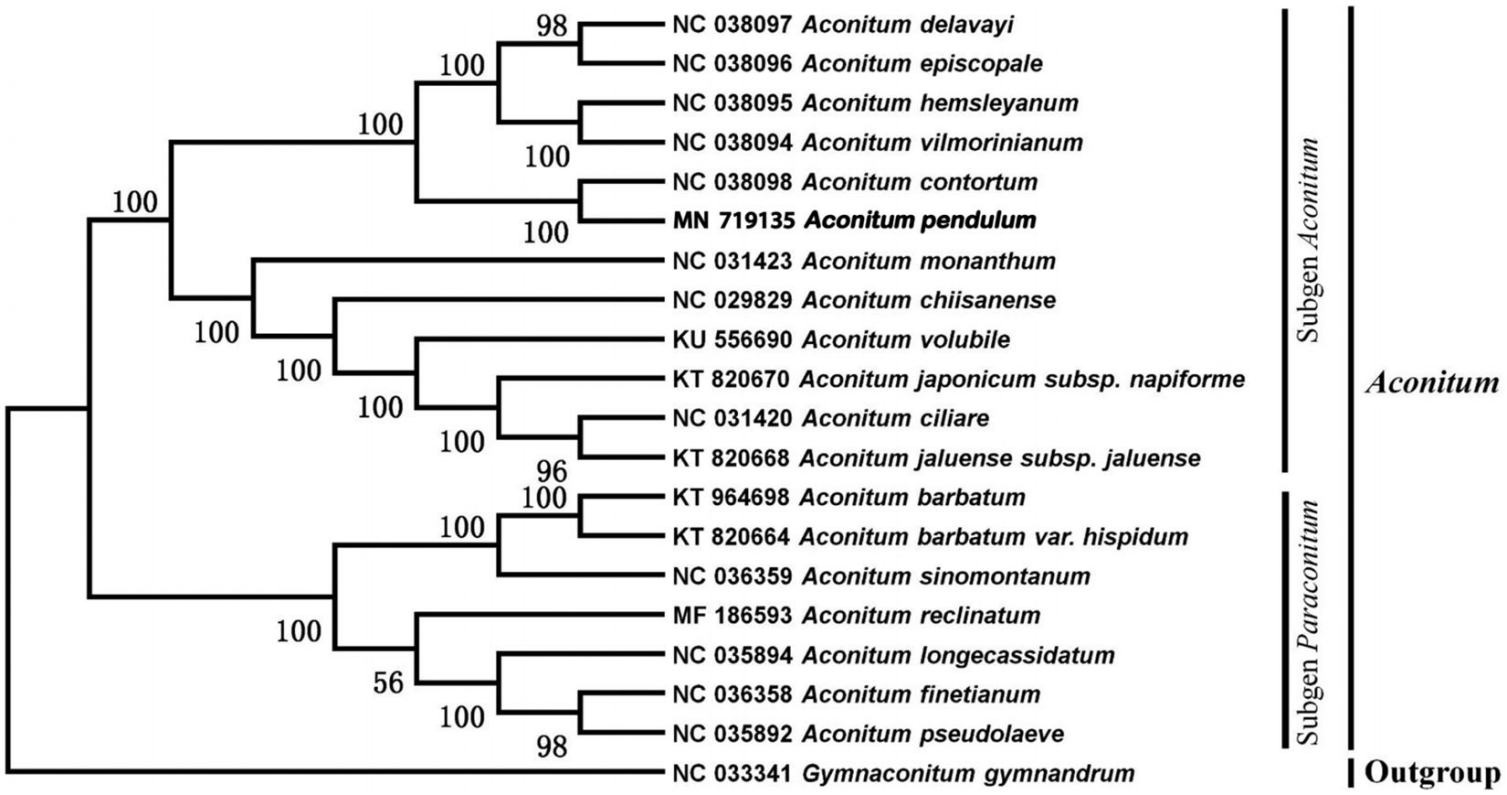
Maximum likelihood phylogenetic tree based on 20 complete chloroplast genomes (bootstrap repeat is 1000).
